# Near Add Power of Glaucoma Patients with Early Presbyopia

**DOI:** 10.3390/jcm13195675

**Published:** 2024-09-24

**Authors:** Masahiko Ayaki, Kazuo Ichikawa

**Affiliations:** Chukyo Eye Clinic, Nagoya 456-0032, Japan; ichikawa@chukyogroup.jp

**Keywords:** presbyopia, glaucoma, near add power, FP receptor agonist, ocular accommodation

## Abstract

**Purpose:** Glaucoma medication may accelerate the progression of presbyopia. The aim of this study was to compare presbyopia between controls and patients with glaucoma in their 40s. **Methods:** This was a cross-sectional study of bilateral phakic participants aged between 40 and 49, which included controls (*n* = 114, mean age 46.1 ± 2.7 y) and patients with primary open-angle glaucoma (*n* = 105, 46.4 ± 2.7 y) who had been using FP receptor agonists, beta blockers, and carbonic anhydrase inhibitors for at least six months. We compared the near add power between the two groups. **Results:** The mean near add power and the prevalence of symptomatic presbyopia (near add power ≥ 1.50 D) were 1.16 ± 0.74 D and 42.1% for controls and 1.77 ± 0.71 D (*p* < 0.01) and 79.0% (*p* < 0.01) for glaucoma patients, respectively. The odds ratio (OR) and confidence interval for symptomatic presbyopia were associated with age (1.36, 1.21–1.52), ganglion cell complex thickness (0.96, 0.94–0.99), presence of glaucoma (6.19, 3.13–12.23), and number of glaucoma medications (4.26, 2.42–7.43). Among medications, only FP receptor agonists (5.79, 2.68–12.32) produced significant results. Survival analysis showed that glaucoma patients reached the threshold of a near add power of +1.50 D significantly sooner than controls (*p* < 0.05; log-rank test). **Conclusions:** Glaucoma patients, especially those using FP receptor agonists, had higher near add power than controls.

## 1. Introduction

Presbyopia is the gradual decline in the ability to focus on near objects with age and is a common ocular affliction worldwide [1,2]. As global life expectancy increases, managing presbyopia becomes crucial for maintaining quality of life and independence for affected individuals. The accommodative amplitude, which reaches its maximum of approximately 15 diopters during the teenage years, declines linearly thereafter [3,4,5]. By age 25, approximately half of the maximum accommodative amplitude has already diminished and until the mid-40s, it deteriorates to zero. Accommodation is driven mainly at the lens and ciliary muscle where the lens thickens for near vision and thins for distant vision, which is regulated by the contraction and relaxation of the ciliary muscle. The ciliary muscle is tethered anteriorly to the scleral spur, trabecular meshwork, Schlemm’s canal, and Schwalbe’s line, which is located at the junction of the cornea and trabecular meshwork [6]. With age, lens hardening and ciliary muscle dysfunction can lead to presbyopia.

Glaucoma is a multifactorial optic neuropathy that leads to a decline in visual function in advanced stages [7]. Worldwide, it is a major sight-threatening disease. In Japan, it has a prevalence of 5.0% in the population above 40 years [8]. As a result of visual field defects, the quality of life, including driving performance [9,10], deteriorates. Glaucoma is treated with surgery and topical medication, which is often administered for a lifetime [11]. Many medications effectively suppress the progression of optic nerve damage and visual field defects by reducing intraocular pressure (IOP). FP receptor agonists are the first-line medication for glaucoma treatment; however, they may lead to topical periocular adverse reactions, including dry eye [12], eyelid pigmentation, eyelash changes (e.g., eyelash growth), iris pigmentation, and deepening of the upper eyelid sulcus [13]. Furthermore, recent studies showed that glaucoma medications are associated with surgical failure of trabeculectomy [14] and accelerations of presbyopia progression [15,16,17,18].

A common physiopathology has been suggested for presbyopia and glaucoma, since the ciliary body and ciliary muscle are major components of aqueous production and accommodation, respectively. Additionally, they are typical age-related ocular diseases of an irreversible nature, suggesting a similar pathogenesis. Indeed, several basic and clinical studies support this hypothesis [6,16,17,18,19,20]. Specifically, prostaglandin analogues (PGs) have a contractile effect on the ciliary muscle, as indicated by organ culture experiments [20] that demonstrate a pressure reduction through this action [6,19]. Numerous clinical studies have suggested a reduction in accommodative amplitude with latanoprost [16,17,18] and recent research has revealed that glaucoma patients treated with antiglaucoma eyedrops reached a specific near add power significantly earlier than controls [17,18].

In primary open-angle glaucoma, increased outflow resistance in the conventional pathway occurs due to a high-rigidity trabecular meshwork and excess extracellular matrix proteins [21,22]. Peripheral muscular tendon fibers attach to the trabecular meshwork and affect aqueous outflow during contraction and relaxation [23]. PGs enhance matrix metalloproteinase production, remodeling the extracellular matrix of the ciliary muscle and sclera, increasing uveoscleral outflow and decreasing intraocular pressure. A reduction in the ciliary muscle extracellular matrix reduces hydraulic resistance, contributing to increased uveoscleral outflow [19]. If ciliary muscle attachments are severed, the old muscle can contract as it would in a young eye. The age-related loss of elasticity in posterior attachments suggests presbyopia might be partly due to reduced ciliary muscle mobility [21,23].

A previous study [18] compared the near add power of glaucoma and control patients and identified age, astigmatic errors, mean deviation, ganglion cell complex (GCC) thickness, and retinal nerve fiber layer (RNFL) thickness as significant risk factors for higher near add power.

The purpose of this study was to compare the frequency of ocular symptoms and the near add power between individuals with medically managed glaucoma and a control group in the early stages of presbyopia. Additionally, we sought to identify which specific glaucoma medication significantly affected near add power using regression analysis. Unlike previous studies, this study focused on detecting early presbyopia by analyzing participants aged between 40 and 49 years, an age range in which the relationship between age and accommodation amplitude is roughly linear [3,4,5] and most individuals become aware of focusing difficulties and first use reading glasses. This approach allows us to compare glaucoma and control groups at an earlier age and identify potential factors contributing to the progression of presbyopia. 

## 2. Methods

### 2.1. Study Design, Patient Recruitment and Institutional Review Board Approval

This research was a hospital-centered, cross-sectional cohort study carried out between April 2015 and March 2023. The participants were outpatient cases and were consecutively enrolled from Otake Eye Clinic in Kanagawa, Japan, during the period from December 2018 to March 2023. The Kanagawa Medical Association’s institutional review boards and ethics committees (approval granted on 12 November 2018, under permission number krec2059006) sanctioned the study, which adhered to the principles outlined in the Declaration of Helsinki. Informed consent was not required, as the boards approved an opt-out consent process for this study. The protocol was registered with the UMIN Clinical Trials Registry (UMIN000051891) on 15 August 2023.

### 2.2. Inclusion and Exclusion Criteria 

We consecutively recruited patients aged 40 to 49 years with bilateral phakic eyes and a best-corrected visual acuity of 20/25 or better in both eyes. Patients who had near add power measured were selected and analyzed after classification by control and glaucoma. Exclusion criteria were macular diseases and glaucoma surgery. 

### 2.3. Patient Interviews for Common Eye Symptoms

Participants were asked whether they experienced six common visual symptoms, including eye strain, blurred vision, photophobia, dryness, irritation, and pain, with a yes/no response format. These questions were drawn from the Dry Eye–Related Quality-of-Life Score questionnaire [24] and reflect the most frequently reported symptoms in outpatient eye clinics at Keio University Hospital in 2014. The purpose of this survey was to explore whether the prevalence of common ocular symptoms was associated with the severity of presbyopia.

### 2.4. Ophthalmological Examinations and Diagnosis of Glaucoma 

All participants underwent a comprehensive ophthalmologic evaluation, which included: best-corrected visual acuity; a slit lamp examination; IOP measurement; fundus examination; and standard automated perimetry with the Humphrey Visual Field Analyzer Swedish Interactive Threshold Algorithm–Standard 24-2 program (HFA 24-2: Carl Zeiss Meditec, Dublin, CA, USA). Patients with cataracts or other non-glaucomatous ocular conditions that could cause visual field defects were excluded following a basic eye exam. Glaucoma was diagnosed when at least two reliable visual field tests confirmed glaucomatous visual field defects consistent with glaucomatous optic disc changes [25]. Exclusion criteria also included significant media opacity or other intraocular or neurological diseases affecting the visual field. Additionally, eyes with unreliable visual field results (fixation loss > 33%, false-positive > 15%, or false negative > 20%) were omitted from the study. Consequently, patients with primary open-angle glaucoma were enrolled.

### 2.5. Optical Coherence Tomography (OCT)

OCT (RS-3000, software version 1.4.2.1; Nidek, Aichi, Japan) was used to measure macular retinal nerve fiber layer (mRNFL), ganglion cell layer (GCL) + inner plexiform layer (IPL) (GCL/IPL), mRNFL + GCL + IPL [GCC], and full macula thickness of the maps resulting from macular cube scans of a fovea-centered 6 × 6 mm square area. For peripapillary RNFL imaging, raster scanning over a 6 × 6 mm^2^ area centered on the optic disc center was conducted at a scan density of 512 A-scans (horizontal) × 128 B-scans (vertical). Peripapillary RNFL measurements were obtained using a 3.45 mm diameter circle that was automatically centered around the optic disc. The 12 RNFL sectors (each covering 30 degrees) were numbered starting from the 1 o’clock position. In all cases, the scan circle from the peripapillary protocol did not intersect any areas of parapapillary atrophy. OCT images with a quality score below 30 were excluded. All measurements were taken within three months of the most recent visual field exam.

The topical glaucoma medications used were FP receptor agonists: 0.005% latanoprost, a fixed combination of 0.005% latanoprost (Senjyu pharmaceutical Co., Ltd., Osaka, Japan) and beta blocker, 0.0015% tafluprost (Santen pharmaceutical Co., Ltd., Osaka, Japan), and 0.004% travoprost (Alcon Laboratories, Co., Ltd., Tokyo, Japan); and beta blockers: 0.5% timolol (Senjyu) and 2% carteolol (Otsuka pharmaceutical Co., Ltd., Tokyo, Japan), carbonic anhydrase inhibitor (CAI); 1% dorzolamide (Santen), and 0.1% brimonidine (Senjyu) (Table 1).

A six-month period of eye drop use was deemed sufficient, as previous studies demonstrated significant effects after observation periods of just one day and 30 days [26,27]. The evaluation of control participants involved measuring best-corrected visual acuity measurements, autorefractometry, slit-lamp biomicroscopy, funduscopy, IOP measurements with a non-contact tonometer (TonorefTM II, Nidek Co., Ltd., Aichi, Japan) or Goldmann applanation tonometer Takagi-Seiko, Toyama, Japan), OCT, or Humphrey Field Analyzer (Carl Zeiss, Jena, Germany).

The corneal vital staining and fluorescein tear break-up time were assessed according to previously described procedures [28]. The prescribed eye drops for dry eye treatment included 0.1% hyaluronate (Santen), 3% diquafosol (Santen) and 2% rebamipide (Otsuka).

Binocular near add power was determined by a blinded examiner using a Bankoku near-acuity chart (Handaya Inc., Tokyo, Japan) at a distance of 30 cm. After establishing the patient’s distance refractive correction, the minimum additional power required for near acuity better than 20/25 was measured in increments of 0.25 or 0.50 D and recorded as the near add power.

### 2.6. Statistical Analysis

Patients were categorized into 114 controls and 105 patients with glaucoma based on the specified inclusion and exclusion criteria. Patient demographics and ophthalmological parameters are presented as the mean ± standard deviation for continuous variables and as percentages for categorical variables. *t*-tests and chi-squared tests were used to compare these demographics, as appropriate. To explore possible ophthalmic parameters that were associated with near add power, we performed univariable regression analysis. Consequently, we selected age, spherical equivalent, anisometropia, GCC thickness, peripapillary RNFL thickness, presence of glaucoma and number of glaucoma medications as explained variables. We then estimated the odds ratios (ORs) with 95% confidence intervals for the presence of symptomatic presbyopia (characterized by the cutoff point of near add power [<1.50 D]) in relation to each selected ophthalmic parameter using logistic regression models. Kaplan–Meier survival analysis was used to compare the age at which participants reached a near add power of +1.50 D between control and glaucoma. Results were analyzed with the log-rank test. Given that the decline in accommodation amplitude starts at birth, a near add power of +1.50 D was set as the endpoint at which many people become aware of focusing difficulties. If presbyopia progressed rapidly, the rate decreased earlier. This method has been repeatedly used in references [17,18]. All analyses were performed using StatFlex (Version 7, Atech, Osaka, Japan), with *p* < 0.05 considered to indicate a significant difference.

## 3. Results

FP receptor agonists were used in 87 patients, beta blockers in 37, carbonic anhydrase inhibitors in 13, and brimonidine in 8 patients. A fixed combination of FP receptor agonists/beta blocker or beta blocker/carbonic anhydrase inhibitor was used in 21 patients. The mean number of glaucoma medications was 1.2 ± 0.5. Brimonidine was prescribed as an add-on for eight advanced cases; six patients were prescribed an FP receptor agonist and two patients a fixed combination of FP receptor agonist/beta blocker. The mean age of patients prescribed brimonidine was 45.6 ± 3.4 years, the mean deviation was −8.5 ± 5.9 and the near add power was 2.25 ± 0.65 D. 

**Figure 1:**
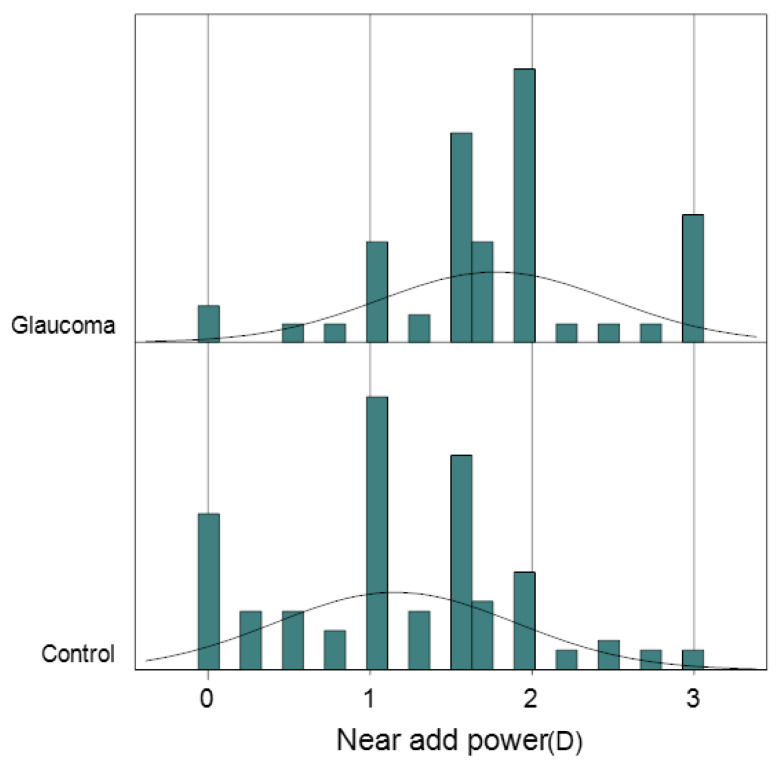
Distribution of near add power with theoretical line. Note how the near add power of glaucoma patients tends to concentrate more in the higher regions compared with control.

The groups did not differ significantly in terms of age, gender, astigmatism, or anisometropia. However, the glaucoma group had a higher degree of myopia, greater near add power, and a higher prevalence of symptomatic presbyopia compared to the control group (Table 2, Figure 1). For glaucoma-related clinical features, all parameters were worse in the glaucoma group except for IOP and full macular thickness. The glaucoma group showed a higher prevalence of dry eye medication use.

Univariable regression analysis of near add power and ocular parameters indicated that age, anisometropia, GCC thickness, and RNFL thickness were significantly associated with near add power (Table 3). Sex was not associated with near add power. Among the categorized glaucoma medication, FP receptor agonist and brimonidine were significantly associated with near add power. Specifically, latanoprost, tafluprost, travoprost, and brimonidine were associated with near add power. Dry eye-related parameters were not associated. 

Comparison of ORs for risk factors of symptomatic presbyopia (near add power ≥ 1.50 D) revealed the OR for symptomatic presbyopia was significant for age, cup/disc ratio, GCC thickness, presence of glaucoma, and number of glaucoma medication (Table 4). Among the categorized glaucoma medication, the OR was significant for FP receptor agonist and brimonidine. Specifically, latanoprost (OR 5.30) and tafluprost (OR 8.62) were significant risk factors. 

**Figure 2:**
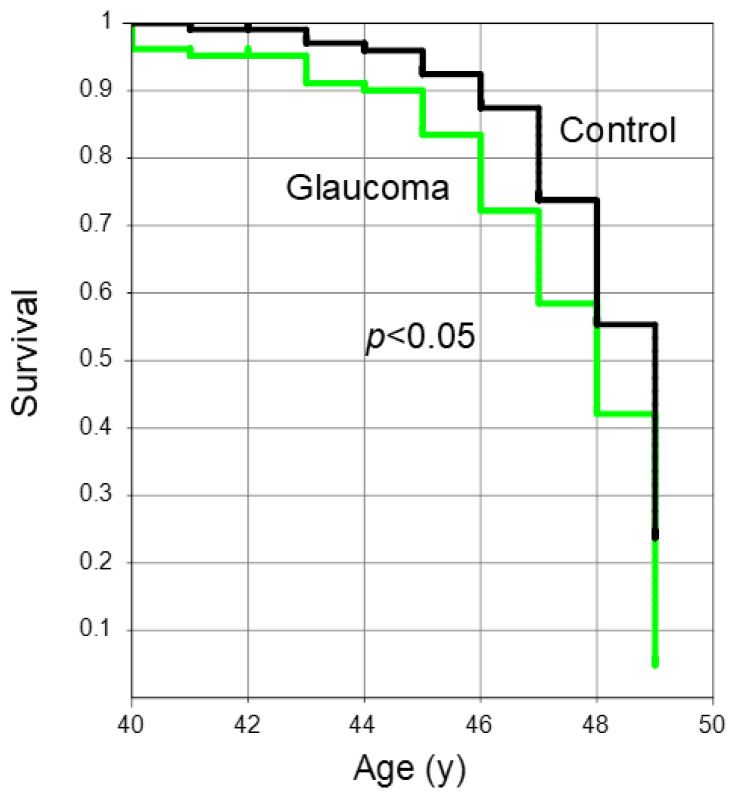
A Kaplan–Meier survival plot showing the age at which individuals in the two groups reached the near add power endpoint of +1.50 D. Glaucoma patients (green) reached the endpoint of +1.50 D significantly earlier than those in the control (black) (*p* < 0.05, log-rank test).

A Kaplan–Meier survival curve illustrated the age at which participants in the two groups attained the near add power threshold of +1.50 D. Those with glaucoma achieved this endpoint significantly sooner compared to the control group (Figure 2, *p* < 0.05, log-rank test).

Prevalence of symptom was not different between the two groups (Table 5).

## 4. Discussion

The current results further confirmed the progression of presbyopia in individuals using different glaucoma medications in early presbyopia. Specifically, users of FP receptor agonists experienced significant effects in terms of presbyopia progression, while those using beta blockers and CAIs did not exhibit such effects (although a previous study suggested beta blockers had some effect on the ciliary muscle [15]). These results aligned with previous investigations that highlighted the exacerbating impact of glaucoma medications on presbyopia by increasing near add power. However, it is essential to interpret these results cautiously. The study population was heterogeneous, with FP receptor agonists being the primary therapy for 82.9% (87/105) of patients. The precise strength of these effects remains inconclusive. Similarly, the effect of brimonidine is debatable, as it was prescribed as an add-on to FP receptor agonists in patients with advanced glaucoma. For early presbyopes with jobs requiring intense accommodation (such as drivers, e-sports gamers and athletes), cautious prescription of FP receptor agonists may be advisable. Alternatively, beta blockers or CAIs could be considered if feasible. Basic research suggested that bimatoprost had the most substantial effect on ciliary muscle contraction [20,29,30]. It could be speculated that continuous muscle contraction due to FP receptor agonists may lead to decreased accommodative amplitude.

The prevalence of symptoms did not differ between the two groups. While significant symptoms can arise from decreased visual function in advanced glaucoma and side effects of glaucoma medications, early glaucoma cases in patients in their 40s are often treated with monotherapy, minimizing adverse effects as indicated previously [31]. Consequently, symptomatic presentation may be less pronounced. In other words, presbyopia remains challenging to identify even with rapidly progressive near add power since patients simply did not distinguish age-related changes from glaucoma-related changes and may have been unaware of a worsening.

The present findings indicate that glaucoma patients were prescribed more dry eye medication compared to the control group. However, there were no significant differences in ocular surface parameters or the prevalence of symptoms between the two groups. It is essential to recognize that both the pharmaceutical components and preservatives in glaucoma medications can lead to various adverse effects [12,32]. Benzalkonium chloride, commonly contained in topical glaucoma medications, has been extensively studied for its side effects [32]. Patients might often desire eye lubricating solutions due to sensitization caused by glaucoma eye drops [12].

This study has several limitations. The pupillary diameter, accommodation amplitude, and corneal topography should have been examined since they are critical elements of presbyopia. Detailed visuals of glaucoma would also help us to assess presbyopia in glaucoma. The current results should be cautiously interpreted due to the heterogeneity of the cohort, in which FP receptor agonist was used in most cases. However, this study successfully identified a significant contribution of FP receptor agonists to the progression of near add power. Another limitation is that the present study does not consider lifestyle factors like screen time or reading habits that may contribute to presbyopia. Medical professionals treating patients with early presbyopia and glaucoma would benefit from detailed recommendations or guidelines in the future. Extensive studies that track individuals over an extended period might be beneficial for validating the results and investigating the long-term consequences of various glaucoma therapies on the advancement of presbyopia.

In conclusion, this study revealed that near add power increased exclusively with FP receptor agonists in glaucoma patients during the age of early presbyopia, whilst this effect was not observed with beta blockers or CAIs. For specific workers requiring intense accommodation, cautious prescription of FP receptor agonists may be advisable.

## Figures and Tables

**Table 1:** Glaucoma medications.

Glaucoma Medications	Number of Cases
FP receptor agonists	77
Latanoprost	53
Tafluprost	13
Travoprost	7
Bimatoprost	4
Beta blockers	16
Timolol	7
Carteolol	9
Fixed combinations	21
Latanoprost/timolol	5
Latanoprost/carteolol	5
Timolol/dorzolamide	11
Dorzolamide	2
Brimonidine *	8 *

* added to FP receptor agonist in all cases.

**Table 2:** Patient demographics and ophthalmological parameters.

	Control	Glaucoma	*p*-Value
Number (% men)	114 (35.1)	105 (44.3)	0.07
Baseline Characteristics			
Age, y	46.1 ± 2.7	46.4 ± 2.7	0.28
Spherical equivalent, D	−3.44 ± 3.30	−4.64 ± 3.42	<0.05
Astigmatism, D	0.34 ± 0.60	0.41 ± 0.64	0.40
Anisometropia, D	0.63 ± 0.73	0.69 ± 0.69	0.54
Near add power, D	1.16 ± 0.74	1.77 ± 0.71	<0.01
Symptomatic presbyopia (near add power ≥ 1.5 D), %	42.1	79.0	<0.01
Glaucoma-related clinical features			
Intraocular pressure, mmHg ^†^	16.1 ± 3.5	15.7 ± 3.3	0.47
Mean deviation, dB ^†^	−2.0 ± 2.8	−5.4 ± 5.3	<0.01
Cup/disc ratio ^†^	0.62 ± 0.13	0.76 ± 0.12	<0.01
GCC thickness, μm ^†^	87.7 ± 17.3	78.9 ± 11.5	<0.01
Peripapillary RNFL thickness, μm ^†^	114.8 ± 16.7	93.7 ± 18.0	<0.01
Full macular thickness, μm ^†^	266.7 ± 34.1	256.5 ± 15.9	0.18
Dry eye-related clinical features			
Tear break-up time, s	3.5 ± 2.5	4.0 ± 2.2	0.16
Short tear break-up time (≤5 s), %	73.3	64.0	0.51
Superficial punctate keratitis, %	27.2	30.0	0.68
Use of dry eye medication, %	8.8	17.9	<0.05

Data are presented as mean ± standard deviation unless specified otherwise. ^†^ Worse eye. *p*-value; unpaired *t* test or chi-square test as appropriate. GCC = ganglion cell complex; RNFL = retinal nerve fiber layer.

**Table 3:** Association between near add power and ocular parameters.

Baseline Characteristics and Refractive Status	ß *	*p*-Value
Age in years	0.32	<0.01
Sex (men = 1)	0.10	0.119
Spherical equivalent	−0.21	<0.05
Astigmatism	0.03	0.56
Anisometropia	0.170	<0.01
Glaucoma-related clinical features		
Intraocular pressure ^†^	0.07	0.27
Mean deviation ^†^	−0.08	0.31
Cup/disc ratio ^†^	0.11	0.13
GCC thickness ^†^	−0.25	<0.05
Peripapillary RNFL thickness ^†^	−0.21	<0.01
Full macular thickness ^†^	−0.24	0.07
Number of glaucoma medications	0.34	<0.01
Presence of glaucoma	0.36	<0.01
Duration of glaucoma medication	0.06	0.45
Glaucoma medications: Categorized medication group		
FP receptor agonists	0.28	<0.01
Beta blockers	0.01	0.87
Carbonic anhydrase inhibitors	0.77	0.43
Brimonidine	0.18	<0.01
Glaucoma medications: Itemized medications		
Latanoprost	0.26	<0.01
Tafluprost	0.22	<0.01
Travoprost	0.20	<0.01
Timolol	0.10	0.09
Carteolol	0.09	0.16
Dry eye-related clinical features		
Tear break-up time	−0.06	0.42
Superficial punctate keratitis	0.07	0.28
Use of dry eye medication	0.08	0.17

* Standardized partial regression coefficient, adjusted for age and sex. ^†^ Worse eye. GCC = ganglion cell complex; RNFL = retinal nerve fiber layer.

**Table 4:** Comparison of odds ratios (ORs) for risk factors of symptomatic presbyopia.

Risk Factors	OR	Upper Limit of 95% CI	Lower Limit of 95% CI
Baseline characteristics and refractive status			
Age	1.364 **	1.219	1.528
Spherical equivalent	0.999	0.998	1.000
Anisometropia	1.002	0.998	1.007
Glaucoma-related clinical features			
GCC thickness ^†^	0.968 *	0.942	0.994
Peripapillary RNFL thickness ^†^	0.981	0.961	1.001
Full macular thickness ^†^	0.981	0.956	1.007
Presence of glaucoma	6.196 **	3.138	12.231
Number of glaucoma medications	4.246 **	2.426	7.432
Glaucoma medications			
Categorized medication group			
FP receptor agonists	5.749 **	2.682	12.321
Beta blockers	0.838	0.263	2.671
Carbonic anhydrase inhibitor	2.367	0.347	16.134
Itemized medications			
Latanoprost	5.306 **	2.381	11.826
Tafluprost	8.672 **	1.791	41.985
Timolol	2.617	0.853	8.027
Carteolol	1.196	0.355	4.028

* *p* < 0.05, ** *p* < 0.01; adjusted for age and sex. ^†^ Worse eye. CI = confidence interval; GCC = ganglion cell complex; RNFL = retinal nerve fiber layer.

**Table 5:** Prevalence of symptoms.

Symptoms	Control	Glaucoma	*p*-Value
Dry sensation	26.5	16.8	0.13
Ocular discomfort	14.2	14.9	0.89
Ocular pain	7.1	3.0	0.28
Eye strain	19.5	22.8	0.56
Sensitivity to bright light	9.7	7.9	0.76
Blurring	8.0	8.9	0.68

Values are percentage prevalence.

## Data Availability

The data collected during the current study are available from the corresponding authors upon reasonable request.
